# Making the Psychological Dimension of Learning Visible: Using Technology-Based Assessment to Monitor Students’ Cognitive Development

**DOI:** 10.3389/fpsyg.2019.01368

**Published:** 2019-06-10

**Authors:** Gyöngyvér Molnár, Benő Csapó

**Affiliations:** ^1^Department of Learning and Instruction, University of Szeged, Szeged, Hungary; ^2^MTA–SZTE Research Group on the Development of Competencies, University of Szeged, Szeged, Hungary

**Keywords:** technology-based assessment, online assessment, assessment for learning, visible learning, cognitive development

## Abstract

Technology-based assessment offers unique opportunities to collect data on students’ cognitive development and to use that data to provide both students and teachers with feedback to improve learning. The aim of this study was to show how the psychological dimension of learning can be assessed in everyday educational practice through technology-based assessment in reading, mathematics and science. We analyzed three related aspects of the assessments: cognitive development, gender differences and vertical scaling. The sample for the study was drawn from primary school students in Grades 1–8 (ages 7 to 14) in Hungary. There were 1500 to 2000 students in each grade cohort. Online tests were constructed from 1638 items from the reading, mathematics, and science domains in the eDia system. The results confirmed that the disciplinary, application and psychological dimensions of learning can be distinguished empirically. Students’ cognitive development was the most steady (and effective) in mathematics, where the greatest development occurred in the first years of schooling. Path models suggested that the psychological dimension of learning can be predicted at a moderate level based on students’ level of school knowledge consisting of the disciplinary and application dimensions of learning as latent constructs. The predictive power was almost the same in both dimensions. Generally, girls developed faster in the psychological dimension of reading, mathematics and science learning; however, the size of gender differences varied by age and domain. This study (1) provides evidence that the psychological dimension of learning can be made visible even in an educational context, (2) highlights the importance of the explicit development of the psychological dimension of learning during school lessons, and (3) shows that there are gender differences in the developmental level of the psychological dimension of learning in favor of girls but that this varies by grade and domain.

## Introduction

Improving students’ cognitive abilities has always been a goal of schooling since the very beginning of formalized education (Hattie and Anderman, 2013). However, despite the theoretical foundations, assessment instruments and pedagogical practices that have evolved over time, this aim has not yet been met; in many school systems students’ cognitive abilities are not optimally enhanced. In the 20th century, several research schools and paradigms sought to conceptualize cognition, define its key constructs and make them measurable (see e.g., Binet and Simon, 1916; Inhelder and Piaget, 1958; Adey, 2007). Among these, research on intelligence and the related psychometric tradition, Piaget and his school, and the cognitive revolution have all had a major impact on redefining the goals of education. The implications of the research within these paradigms were drawn for educational practice, and a number of mostly stand-alone programs were initiated in the 1970s, outside classroom instruction (Feuerstein et al., 1980; Klauer, 1989a,b, 1991, 1993, 1997). Later on, in the 1990s, developmental effects were embedded in school subjects using the content of learning (Adey and Shayer, 1994; Shayer, 1999; Adey et al., 2001; Shayer and Adey, 2002; Shayer and Adhami, 2007). The related research, including a number of experiments, resulted in a better understanding of the role that cognitive processes play in school learning, but it has had a modest impact on educational practice.

At the beginning of this millennium, more or less the same ideas emerged in a new wave of teaching 21st-century skills. Several projects sought to define, operationalize, measure and teach these skills (see e.g., Trilling and Fadel, 2009; Griffin and Care, 2014), but the same constraints appeared to hinder progress in putting these ideas into practice in mass education as with previous similar attempts. There were no proper tools for assessing and monitoring changes in students’ cognition. The availability of appropriate assessment instruments is a necessary condition for any pre-test – post-test experimental design as well. However, what can be created and applied in specific experimental conditions cannot always be scaled up for broader practical applications. Similarly, the roots of a number of practical educational challenges can be traced back to the fact that significant determinants of school learning are not visible (Hattie, 2009). They are also not easy to observe, nor can developmental deficiencies always be identified by teachers (MacGilchrist et al., 2004). The lack of thinking skills – the cognitive tools required for successful learning – are not identified; thus, they remain untreated, and this significantly hampers further learning.

Thinking, or more specifically, a set of cognitive skills essential for learning, such skills are not observable in the everyday educational context. Students are not aware of the existence of the required processes, and teachers, even if they receive training in identifying the cognitive processes underlying learning, are not able to observe them, or they simply have no time or capacity to determine each student’s individual needs. Although the developmental levels of crucial thinking skills might be measured with traditional paper-based instruments, the immense costs, the human resources required, and the time between assessment and feedback excludes the possibilities of using them diagnostically. Technology may be a solution for making thinking processes visible by creating simpler, faster, frequently applicable and cost-effective assessments (Mayrath et al., 2012).

In this paper, which is part of a larger project, we present the results of work in identifying cognitive processes relevant for learning, making them measurable in normal educational contexts, and providing students and teachers with frequent feedback. One of the most challenging aspects of this work, is establishing the validity of diagnostic instruments to assess of cognitive processes; showing that the tests measure something more than mastering the current teaching material. To do this, we empirically validated a 3-dimensional framework developed for diagnostic assessment and explored the psychometric characteristics of an item bank devised for the assessment of the psychological dimension of learning.

## Theoretical Background

The idea of making learning visible was introduced into educational research and development by John Hattie. He made a great step forward in initiating evidence-based educational practice when he synthesized the results of over 800 meta-analyses (Hattie, 2009). He translated his findings into actual classroom work, and in his book for teachers, he explained:

Visible teaching and learning occurs when learning is the explicit and transparent goal, when it is appropriately challenging, and when the teacher and the student both (in their various ways) seek to ascertain whether and to what degree the challenging goal is attained, when there is deliberate practice aimed at attaining mastery of the goal, when there is feedback given and sought, and when there are active, passionate, and engaging people (teachers, students, peers, and so on) participating in the act of learning (Hattie, 2012, p. 18).

As he emphasizes, feedback plays a central role in successful learning, which at a higher level of learning, includes self-monitoring, self-evaluation and self-assessment. However, he also explains how difficult a task it is to provide proper feedback: “Learners can be so different, making it difficult for a teacher to achieve such teaching acts: students can be in different learning places at various times, using a multiplicity of unique learning strategies, meeting different and appropriately challenging goals” (Hattie, 2012, p. 18).

Student diversity, i.e., students at different levels in different cognitive attributes, is not the most challenging phenomenon when proper feedback is considered. A major problem is that a number of learning outcomes, sometimes the most important ones, are not visible and cannot easily be made visible. While the majority of the studies Hattie reports on deal with organizational issues, methods and classroom practices for teaching curricula, there are far fewer studies that cover the underlying cognitive processes, e.g., reasoning skills, required to understand mathematics and science or precursors of reading, such as phonemic awareness. Some studies have focussed on the most hidden aspects of learning. For example, Ritchhart et al. (2011) identify a broad range of teaching and learning practices to make thinking visible. They identify the crucial problem in a simplified conception of learning (reduced to memorization) and knowledge (reduced to information, facts and figures): “When we demystify the thinking and learning processes, we provide models for students of what it means to engage with ideas, to think, and to learn. In doing so, we dispel the myth that learning is just a matter of committing the information in the textbook to one’s memory” (Ritchhart et al., 2011, p. 28).

Taking into account diversity among students, the limited capacity of teachers and the need to provide feedback on the most relevant but least visible aspects of school learning – promoting students’ cognitive development – we may conclude that students and teachers need a different approach to assessment to improve learning. The online assessment system, eDia, was designed for this purpose. It assesses “thinking,” or “cognitive development,” as a separate dimension, which we call the psychological dimension of learning. We briefly introduce the 3-dimensional theoretical framework that forms the basis for the diagnostic assessment system, and then we elaborate on the psychological dimension in more detail, as that is the focal topic of the present study. Finally, we discuss the crucial role of technology, arguing that its widespread availability in schools makes the time right for such a system to be introduced and integrated into regular educational processes.

### Learning and Cognitive Development: A 3-Dimensional Model of Learning Outcomes

An online diagnostic assessment system, eDia, has been constructed to provide teachers and students with relevant feedback information (Csapó and Molnár, unpublished). The eDia system covers the three most frequently assessed domains of school education; reading, mathematics and science. Large item banks have been developed for use in regular classroom assessments in Grades 1 to 6 of primary school, and for Grades 7 and 8 to explore the developmental trends in a broader age range.

The objectives of each item bank are defined in its assessment framework, similarly to international comparative studies, such as Trends in International Mathematics and Science Study (TIMSS; Mullis et al., 2005) and Progress in International Reading Literacy Study (PIRLS; Mullis and Martin, 2015); they are based on a 3-dimensional model of the goals of learning that forms a common foundation for diagnostic assessment. The three dimensions include thinking/reasoning, application and disciplinary knowledge. [The 3-dimensional framework has been published in several articles and book chapters before the assessment frameworks were elaborated (see e.g., Csapó, 2010; Nunes and Csapó, 2011; Adey and Csapó, 2012; Blomert and Csépe, 2012)]. The framework for reading was somewhat different those for mathematics and science (Csapó and Csépe, 2012; Csapó et al., 2015c), which were more similar (Csapó and Szendrei, 2011; Csapó and Szabó, 2012; Csapó et al., 2015a,b).

The intention of “cultivating the mind” – developing cognitive abilities – may be traced back to ancient philosophy. To set goals in this direction, a model of mind is needed; more specifically, knowledge of how internal psychological attributes are structured and how psychological processes play a role in learning (see more details in the next section). In the eDia frameworks, this is the “thinking” (this term is mostly used in the context of mathematics and science), or, more generally, the “psychological dimension.” According to the model, we propose the psychological dimension of knowledge does not only contain “domain-specific reasoning skills,” but also general reasoning skills embedded in different content and contexts, which has lately been referred to as transversal skills; and is not the same as procedural knowledge. We assume that there are natural cognitive developmental (psychological) processes. These processes, as described by Piaget, take place in the interaction between the child and his/her environment. School education may stimulate this development if it provides a student with proper environmental stimuli and if these stimuli are within the zone of proximal development (ZPD) of the child (Vygotsky, 1978). Very often, school instruction is not adjusted to the individual needs of the students; usually the stimuli are far beyond their ZPD. In these cases, students benefit little from instruction; they memorize the rules and develop specific skills through a large amount of drill practice, which have any real impact on their cognitive development. For example, students may learn rules to deal with ratios and proportions without this learning having much impact on the development of proportional reasoning. Schools may teach students a great deal about combinatorics, probability and correlation without having a real impact on the development of combinatorial, probabilistic or correlative reasoning. In this way, we distinguish the psychological dimension from the disciplinary dimension, which may include procedural knowledge (e.g., skills for solving linear equations or proving geometric theorems) or domain-specific reasoning skills. This model and approach opens the door to fostering domain-general reasoning skills in a domain-specific context.

Application deals with another ancient goal – that school should teach something that is applicable beyond the school context. Applying knowledge and transferring it to new contexts require a deeper conceptual understanding and usually specific exercises to facilitate application. Therefore, most knowledge mastered at school remains inert and not applicable in new contexts (Alexander and Murphy, 1999; Bransford and Schwartz, 1999; Csapó, 2010). The PISA conducted by the OECD has focussed on this dimension from the very beginning. The PISA expert groups elaborated the concept of applicable knowledge and defined it as competencies students need in a modern society. To develop such a framework, the social relevance of knowledge, i.e., the needs of societies have also be taken into account. For the frameworks of the first and second PISA assessment, the concept of literacy was extended in include the objects of the assessment in the three domains as reading literacy, mathematical literacy and scientific literacy (OECD, 1999, 2003).

Disciplinary knowledge is the third dimension and is most commonly known as curricular content. Arts and sciences content constitutes the major source of disciplinary knowledge. The first major international comparative studies (e.g., First and Second International Mathematics Study – Husén, 1967; Burstein, 1993; First and Second International Science Study – Bloom, 1969; IEA, 1988), the precursor to the TIMSS, assessed this dimension. The first assessments were based on an analysis of the curricula in the participating countries. More recently, the TIMSS frameworks organize the objects of the assessment into three groups: content, application and reasoning. This classification bears some similarity to the 3-dimensional eDia frameworks (For PISA assessment frameworks, see OECD, 2003).

Education must not be reduced to providing the right answer quickly, but must deal with the ongoing cognitive work of understanding new ideas and information that will serve students as learners in the future (Costa and Kallick, 2009). In modern society, students are expected to apply their knowledge in a wide range of contexts, and they should be able to solve problems in unknown, novel situations. Thus, these goals must reinforce and interact with each other as they are strongly connected (Molnár and Csapó, 2019).

It is reasonable that the earliest efforts to measure knowledge learnt at school focussed on areas that were the easiest to measure: the disciplinary (knowledge) dimension of learning (see e.g., IEA TIMSS). The goal of applying that knowledge in a new context (the application dimension) and assessing students’ ability to do so is a more complicated task (see e.g., OECD PISA). The goal of developing students’ thinking abilities (the psychological dimension) is even more complex. To be able to make thinking visible, we must be clear about, and draw on, our understanding of what thinking is and what types of thinking we want to assess and enhance.

### Assessment Beyond the Content of Actual Learning

In the 20th century, several research paradigms have conceptualized the development of thinking and its relationship to school education. Among these, research on “intelligence” was the first that was closely linked to education. The first intelligence test (Binet–Simon test, Binet and Simon, 1916) was constructed to assess children’s preparedness for schooling, and the Scholastic Aptitude Test (SAT) (see Grissmer, 2000) served a similar purpose at the transition from secondary to tertiary education. Several new approaches, models and interpretations of the concept of intelligence have been proposed. From the perspective of education, the more useful ones consider intelligence as (able to be modified, taught, learnt, or improved within educational contexts). Our psychological dimension in each domain may thus overlap with the inductive reasoning components of “fluid” intelligence. The psychological dimension can be embedded within the conception of plastic general ability (see Adey et al., 2007), and a number of cognitive skills covered by the psychological dimension of our frameworks are explicitly identified in Carroll’s three-strata model of abilities (Carroll, 1993) and the Specialized Cognitive Systems of Demetriou’s model (Demetriou et al., 1992, 1993; Adey et al., 2007). On the other hand, we emphasize that all the cognitive skills discussed in the psychological dimension of the frameworks are embedded within the content and context of each particular domain, and the tasks developed from the frameworks are adjusted to the developmental level of the cohort of students to be assessed.

The work of Jean Piaget and his school was characterized by another approach. Piaget described students’ reasoning skills with well-defined operations, which correspond with certain mathematical structures (see e.g., Inhelder and Piaget, 1958). He mostly used basic science content for his experiments (e.g., the pendulum), and the operations he identified may be found in various learning contexts as well as in everyday problems.

The cognitive revolution in psychology provided a new impetus to research efforts in school learning. It led to a more differentiated conception of knowledge and learning, allowing a more precise definition of the goals of education. Recent studies in psychology and education have shown that these skills are especially crucial at the beginning years of schooling, as students’ developmental level determines later success (see Nguyen et al., 2016).

The psychological dimension has been conceptualized as the interaction between the development of students’ thinking skills and learning at school (Nunes and Csapó, 2011; Adey and Csapó, 2012; Blomert and Csépe, 2012) and must address how students learn in reading, mathematics and science.

In this study, we explored the prospects of making the psychological dimension of learning visible by using technology-based assessments to monitor the development of students’ thinking skills. The aim of this study was to show how the psychological dimension of learning (thinking) can contribute to the development of specific reasoning skills.

In reading, assessment of the psychological dimension (thinking and reasoning) covers the cognitive mechanisms of development from laborious phonological decoding to the automatic recognition of whole words, and from prerequisite skills of reading through phonemic, phonological and morphological awareness to metacognitive aspects (Blomert and Csépe, 2012). In mathematics (Nunes and Csapó, 2011) and science (Adey and Csapó, 2012), there are generic objects and domain specific objects. For example, number sense is specific to mathematics, while the control of variables and scientific reasoning are better covered within the science framework. Operational reasoning (e.g., seriation, class inclusion, classification, combinatorial reasoning, probabilistic reasoning, proportional reasoning) and some higher-order thinking skills (e.g., inductive reasoning and problem solving) are more generic and can be assessed in both mathematics and science.

## Aims, Research Questions and Hypotheses

In this study, we explored the prospects of making the psychological dimension of learning visible by using technology-based assessments to monitor the development of students’ reasoning skills. The aim of the study was to show how the psychological dimension of learning (thinking) can be assessed in everyday educational practice and how it is related to students’ level of subject matter content knowledge. Three domains were explored from this perspective: reading, mathematics and science. Reading is the basis for all further learning, including mathematics and science, while mathematics provides foundations for learning in various areas of science. These domains are central in many education systems, and large-scale international comparative studies, such as TIMSS, PIRLS, and PISA, have focussed on these areas. We analyzed three aspects of the assessments: cognitive development, gender differences and vertical scaling.

Worldwide, there are many initiatives and computer-based tests available in the domains of reading, mathematics and science worldwide. However, they mainly focus only on disciplinary knowledge dimension (content) or the application dimension (literacy of learning) (e.g., TIMSS, PIRLS, and OECD PISA). There are no regular large-scale assessments that include the psychological dimension of learning in primary school – cognitive development. The available assessment systems in reading, mathematics and science have been designed to assess older students’ reading, mathematics and science knowledge (e.g., TIMSS, PIRLS, and PISA). The present study sought to: (1) define and examine the different dimensions of learning in reading, mathematics and science; (2) monitor and compare cognitive development (the psychological dimension of learning) in the three domains over time; (3) analyze the proportion of unexplained variance in cognitive development if school knowledge (the application and disciplinary dimensions) is taken into account in reading, mathematics and science; and (4) identify any gender differences in the cognitive development in the three domains. We sought to answer five research questions.

RQ1: Can the three dimensions of learning be distinguished empirically? We explored this question to see if cognitive development, the development of reasoning skills, can be assessed separately and be made visible in everyday educational practice. We hypothesize that the psychological, application and disciplinary dimensions of learning can be distinguished empirically, assessed and monitored in everyday educational practice (Csapó and Szendrei, 2011; Csapó and Csépe, 2012; Csapó and Szabó, 2012). We also hypothesize that they will interact and correlate with each other.

RQ2: Is the psychological dimension of learning the same across the three domains? That is, is the same construct being measured in the psychological dimension of learning across the three main domains? The roots of cognitive development may be universal as early neurocognitive development in children is similar across cultures and societies (Molnár and Csapó, 2019). Therefore, based on the conceptualization of the psychological dimension of learning as the interaction between students’ cognitive development and learning at school (Nunes and Csapó, 2011), we hypothesize that the 1-dimensional model will fit the data better than the 3-dimensional model. However, we argue that the 3-dimensional model will take into account results from research on knowledge transfer. According to McKeachie (1987), “Spontaneous transfer is not nearly as frequent as one would expect” (p. 709).

RQ3: How does the psychological dimension of reading, mathematics and science develop over time during primary schooling? Based on previous research results on reasoning skills, we hypothesize that children’s cognitive development is slow (Molnár et al., 2013, 2017), indicating the need for more stimulating school lessons. Based on Polya’s (1981) theory of problem solving, and results from research on mathematics teaching (e.g., Nunes and Csapó, 2011), we hypothesize that the psychological dimension of learning in mathematics will develop the most readily.

RQ4: How can the psychological dimension of learning be explained by students’ level of school knowledge in reading, mathematics and science? That is, how can learning in reading, mathematics and science contribute to the development of the psychological dimension of learning, and how effectively does it stimulate students’ general cognitive development? Research in this field provides rich resources ranging from the classical work of Piaget (see e.g., Inhelder and Piaget, 1958) to the most recent neurocognitive studies (such as Geake and Cooper, 2003; Thomas et al., 2019). We hypothesize that learning reading, mathematics and science will contribute to students’ development in the psychological dimension of learning but that the transfer effect will be low. We base our hypothesis on empirical research that has found that reasoning skills develop relatively slowly during primary and secondary education with the average pace of development being about one quarter of a standard deviation per year (Csapó, 1997; Molnár and Csapó, 2011; Greiff et al., 2013; Molnár et al., 2013, 2017). The development of reasoning skills is a “by-product” of teaching rather than guided by explicit instruction (de Koning, 2000).

RQ5: How does the developmental level of the psychological dimension of learning differ by gender, grade and domain? Based on the most prominent international studies (Martin et al., 2016; Mullis et al., 2016, 2017; OECD, 2016) and the research results on gender differences in students’ development of reasoning skills (Wüstenberg et al., 2014), we hypothesize gender differences in the development of the psychological dimension of learning will vary by grade and domain. The PISA studies indicated that the achievement of 15-year-old Hungarian girls in the application dimension of reading was significantly better than that of boys, while there were no statistically significant gender differences in mathematics and science (OECD, 2016). In contrast, the TIMSS studies that focus on younger students (Grades 4 and 8; 10- to 14-year-olds) mainly assess the disciplinary dimension of mathematics and science knowledge. Their findings indicated that boys significantly outperform girls in mathematics in Grade 8 (Mullis et al., 2016), but there was no statistically significant gender difference in Grade 4. In science, boys significantly outperformed girls at both grade levels (Martin et al., 2016). In PIRLS, Grade 4 Hungarian girls significantly outperformed their boys in reading (Mullis et al., 2017). Please note that the present study focussed on the psychological dimension and not on the application or disciplinary dimensions of learning in mathematics, science or reading.

## Materials and Methods

### Participants

The sample of students for the study was chosen from the partner school network of the Center for Research on Learning and Instruction at the University of Szeged in Hungary. As schools participated voluntarily in the project, representative sampling of school classes or students was not a goal. However, based on the data collected from the schools, it was possible to generate nationally representative indicators for the main variables. We noted that schools with relatively large numbers of low socioeconomic (SES) students were under-represented in the present study, possibly due to the lack of ICT available in those schools.

The sampling unit was a school class. Classes were drawn from primary and secondary schools from Grades 1–8 (aged 7–14). A total of 656 classes from 134 schools in different regions were involved in the study, resulting in a wide-ranging distribution of students’ background variables. The total number of students involved in the study was 14,062 (Table 1). The proportion of boys and girls was about the same. As participation was voluntary, not all students completed tests in all three domains or in each dimension within each domain. Thus, data was potentially available for students who completed nine elements: the assessment of three dimensions of learning (psychological, application, and disciplinary) in three domains (reading, mathematics, and science). After the scaling procedure, we excluded students from the analyses where, because of missing data. it was not possible to compute an ability level in at least one of the nine elements. Thus, 5,714 students from 310 classes and 97 schools were involved in the analyses.

**Table 1:** The sample for the study.

Grade	Whole sample			Data analyzed (3 domains × 3 dimensions)		
	N	Age [mean (SD)]	Gender (% of girls)	N	Age [mean (SD)]	Gender
1	1003	7.8 (0.58)	47.2	349	7.8 (0.59)	46.3
2	1348	8.8 (0.61)	51.5	528	8.8 (0.57)	49.6
3	1675	9.8 (0.62)	49.9	598	9.8 (0.65)	49.9
4	2148	10.8 (0.60)	50.2	659	10.8 (0.60)	49.3
5	2441	11.8 (0.60)	47.8	1169	11.8 (0.61)	47.8
6	2122	12.9 (0.59)	47.7	1017	12.9 (0.59)	47.0
7	1875	13.9 (0.62)	49.6	800	13.9 (0.61)	50.0
8	1450	14.9 (0.63)	49.5	594	14.9 (0.63)	49.7
Total	14062	11.6 (2.18)	49.1	5714	11.7 (2.15)	49.0


### Tests

An item bank was constructed for diagnostic assessments in reading, mathematics and science based on the three dimensions of learning described in the previous section. These item banks collectively contained almost 17,000 tasks with most tasks having several items. There were 6685 tasks for reading, 6691 for mathematics and 3535 for science. Tests to measure the psychological, application, and disciplinary dimensions of learning in reading, mathematics and science among students in Grades 1–6 (aged 6–7 to 12–13). The tests for the study were drawn from these item banks. Students in Grades 7 and 8 received tasks originally written for students in Grades 5 and 6 (see Table 2).

**Table 2:** The structure of the tests in mathematics by cluster of tasks for each grade level.

Grade	Cluster 1	Cluster 2	Cluster 3	Cluster 4	Cluster 5
1	Mouse usage warm-up tasks	MD1 (15)	MR1 (15)	MA1 (15)	MD2/MR2/ MA2 (10)
		MA1 (15)	MD1 (15)	MR1 (15)	MD2/MR2/ MA2 (10)
		MR1 (15)	MA1 (15)	MD1 (15)	MD2/MR2/ MA2 (10)
2	Mouse usage warm-up tasks	MD1 (15)	MR2 (15)	MA2 (15)	MD3 (10)
		MA1 (15)	MD2 (15)	MR2 (15)	MA3 (10)
		MR1 (15)	MA2 (15)	MD2 (15)	MR3 (10)
3	MD1 (10)	MD2 (10)	MA2 (15)	MR3 (15)	MD4 (10)
	MA1 (10)	MA2 (10)	MR2 (15)	MD3 (15)	MA4 (10)
	MR1 (10)	MR2 (10)	MD2 (15)	MA3 (15)	MR4 (10)
4	MD2 (10)	MD3 (10)	MA4 (15)	MR4 (15)	MD5 (10)
	MA2 (10)	MA3 (10)	MR4 (15)	MD4 (15)	MA5 (10)
	MR2 (10)	MR3 (10)	MD4 (15)	MA4 (15)	MR5 (10)
5	MD3 (15)	MD4 (15)	MA5 (20)	MR5 (20)	MD6 (15)
	MA3 (15)	MA4 (15)	MR5 (20)	MD5 (20)	MA6 (15)
	MR3 (15)	MR4 (15)	MD5 (20)	MA5 (20)	MR6 (15)
6	MD4 (15)	MD5 (15)	MA6 (20)	MR6 (20)	MD6 (15)
	MA4 (15)	MA5 (15)	MR6 (20)	MD6 (20)	MA6 (15)
	MR4 (15)	MR5 (15)	MD6 (20)	MA6 (20)	MR6 (15)
7–8	MD5 (15)	MR5 (15)	MA5 (20)	MD6 (20)	MR6 (15)
	MA5 (15)	MD5 (15)	MR5 (20)	MA6 (20)	MD6 (15)
	MR5 (15)	MA5 (15)	MD5 (20)	MR6 (20)	MA6 (15)


*M, mathematics; D, disciplinary dimension; A, application dimension; R, reasoning dimension; 1–6, grade for which the task was originally designed; (NUMBER), number of items in the cluster*.

For each grade level, nine tasks with different difficulty levels (three easy, three medium-difficulty and three difficult) were chosen from each item bank to assess each dimension. After this procedure, there were 543 tasks in reading, 604 in mathematics and 492 in science.

The tasks were grouped into clusters, with 10–15 items per cluster for students in the lower grades and 15–20 items for students in the higher grades. One 45-min test consisted of four clusters of tasks for students in Grades 1 and 2 (50–55 items) and five clusters for students in Grades 3 to 6 (60–85 items). Each test contained clusters of tasks from each learning dimension with the clusters positioned in a different order to avoid the item-position effect in the scaling procedure. Anchor items were used within and between the different grades for the horizontal and vertical scaling of the data. The clusters contained easier or harder tasks from lower or higher grades. A total of 483 strongly anchored, but different clusters were developed from the items selected.

For optimizing the measurement error of the test, the clusters contained tasks from the same dimension of learning, ranging in task difficulty for the different grade levels. That is, students received more tasks from one learning dimension if those tasks were originally prepared for students in lower or higher grades. The structure of the test of mathematical knowledge is presented in Table 2 paralleled the structure of the reading and science tests. Based on this structure, 162 different tests (nine in each grade and each domain) were constructed from the item banks for the vertical scaling of students in Grades 1–8.

In Grades 1–3, instructions were provided in written form, on-screen, and with a pre-recorded voiceover to avoid any reading difficulties and to ensure greater validity of the assessments. Thus, students used headphones during the administration of the tests. After listening to the instructions, they indicated their answer by using the mouse or keyboard (in the case of desktop computers, which are most commonly used in Hungarian schools) or by directly tapping, typing or dragging the elements of the tasks using their fingers on tablets.

**FIGURE 1:**
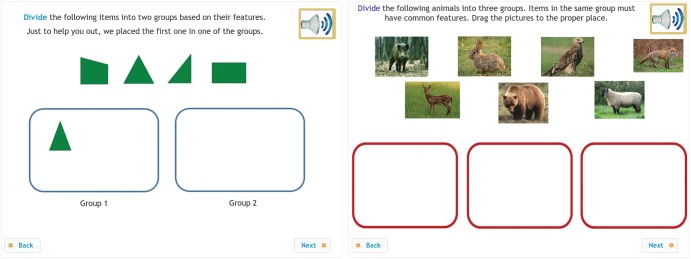
Measuring the psychological dimension of learning: assessment of students’ inductive reasoning skills in the context of geometry and biology.

The tasks presented in Figure 1 assess students’ mathematical and scientific reasoning. Based on the framework for the diagnostic assessment of mathematics (Csapó and Szendrei, 2011) and science (Csapó and Szabó, 2012), the main questions in this psychological dimension related to how well mathematics and science education was adjusted to students’ psychological development, how learning mathematics and science could contribute to the development of specific reasoning skills and how effectively they could stimulate students’ general cognitive development. Items developed to measure the psychological dimension of learning encompassed a long list of skills, such as inductive reasoning, deductive reasoning, analogical reasoning, combinatorial reasoning, systematization skills, proportional reasoning and correlative reasoning. Two examples of tasks for assessing students’ inductive reasoning are presented in Figure 1. Students had to discover regularities by detecting dissimilarities with respect to attributes of different objects. They completed the tasks by dragging the elements to different areas, thereby defining the proper sets. The scoring of all tasks was automated, including items with several correct answers.

**FIGURE 2:**
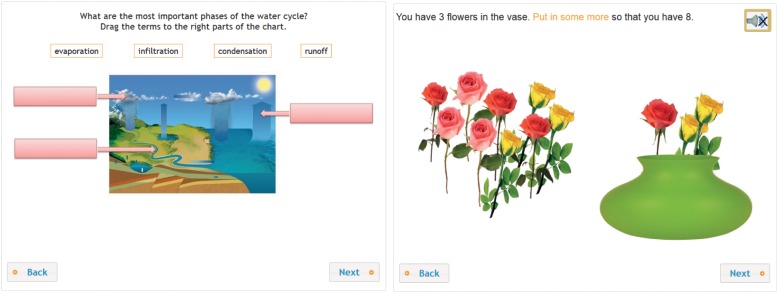
Measuring the disciplinary dimension of learning science and the application dimension of learning mathematics.

Figure 2 presents a task measuring student’s science disciplinary knowledge and a mathematics tasks measuring the application dimension. In the science task, students retrieve disciplinary knowledge of phases of the water cycle. In the mathematics task, students have to select and place flowers – drag and drop – in the vase; only the number of flowers counts. The task measures the application of adding up to 10 in a realistic application context.

### Procedures

The tests were administered over a period of 7 weeks in computer rooms within the participating schools during regular school hours. Each test lasted approximately 45 min. Test sessions were supervised by teachers who had been thoroughly trained in test administration. The tests were delivered on the eDia online platform. After students entered the system and chose the domain (reading, mathematics, or science), the system randomly selected a test for that student from the nine tests available in the appropriate grade level.

To learn to use the program, students were provided with instructions and a trial (warm-up) task with immediate feedback. This instruction included: (1) a yellow bar at the top of the screen to show how far along they were on the test; (2) they had to click on the speaker icon to listen to the task instructions; (3) they had to click on the “next” button to move on to the next task; (4) pupils in Grades 1 and 2 received extra warm-up tasks to enhance keyboarding and mouse skills; and (5) after completing the last task, participants received immediate visual feedback with a display of 1 to 10 balloons, where the number of balloons was proportionate to their achievement.

The feedback system available for the teacher was more elaborate. Due to the large number of students and items, the Rasch analyses were run with the built-in analytic module in the eDia system. As the tasks in the item bank were scaled using IRT, it was possible to compare students’ achievement. Teachers received feedback on students’ achievement both as a percentage of correct items and as ability scores. For each grade and domain, the national average achievement (ability score) was set at 500 with a standard deviation of 100 (Carlson, 2009; Ferrão et al., 2015; Weeks, 2018). This was the point of reference for interpreting students’ achievement.

We used confirmatory factor analyses (CFA) within structural equation modeling (SEM) (Bollen, 1989) to test the underlying measurement models of reading, mathematics and science knowledge in the three dimensions of learning: psychological (reasoning), application (literacy), and disciplinary knowledge, respectively (RQ 1). We used the preferred estimator for categorical variables; the adjusted weighted least squares mean and variance (WLSMV) (Muthén and Muthén, 2012). We tested a 3-dimensional model to distinguish the three different dimensions of learning, and we also tested a 1-dimensional model with all three dimensions combined under one general factor. In order to test which model fitted the data better, we carried out a special χ^2^-difference test in Mplus. We also used CFA to test the underlying measurement model, and to determine the invariance behavior of the psychological dimension across the three domains of learning (RQ 2).

To establish a developmentally valid scale, we used the Rasch model with the vertical and horizontal scaling of the data (RQs 2 and 4) and then a linear transformation of the logit metric. As indicated above, for each domain and at each grade level, the mean achievement of each dimension was set to 500 with a standard deviation of 100. We used path models to test the effect and predictive power of school learning on the psychological dimension of learning (RQ 3).

## Results

### The Psychological Dimension of Learning

Results showed that the psychological (reasoning/thinking), application and disciplinary dimensions of learning can be distinguished empirically and are independent of domain and grade. The χ^2^-difference test in Mplus showed that the 3-dimensional model fitted significantly better than the 1-dimensional model in each grade and in each domain (see Tables 3–5 for reading, mathematics and science, respectively). Generally, the 3-dimensional measurement model for each domain showed a good model fit (Tables 3–5), based on Hu and Bentler’s (1999) recommended cut-off values. The comparative fit index (CFI) and the Tucker–Lewis index (TLI) values above 0.95 and the root mean square error of approximation (RMSEA) below 0.06 indicated a good global model fit.

**Table 3:** Goodness of fit indices for testing the dimensionality of reading from Grades 1 to 8.

Grade	Model	χ^2^	df	p	Δχ^2^	Δdf	p	CFI	TLI	RMSEA	90% C.I.
1	3-dim.	378.262	296	0.001	29.055	3	0.001	0.947	0.941	0.057	[0.038, 0.073]
	1-dim.	448.137	299	0.001				0.903	0.895	0.076	[0.061, 0.090]
2	3-dim.	514.018	461	0.001	30.963	3	0.001	0.975	0.973	0.032	[0.026, 0.075]
	1-dim.	575.196	464	0.001				0.948	0.945	0.047	[0.033, 0.059]
3	3-dim.	406.497	347	0.01	15.681	3	0.01	0.833	0.818	0.054	[0.026, 0.075]
	1-dim.	430.585	350	0.01				0.773	0.755	0.062	[0.039, 0.082]
4	3-dim.	592.821	431	0.01	90.820	3	0.001	0.937	0.932	0.066	[0.052, 0.079]
	1-dim.	695.499	434	0.01				0.898	0.891	0.084	[0.072, 0.095]
5	3-dim.	2046.006	125	0.001	92.737	3	0.001	0.911	0.908	0.035	[0.027, 0.041]
	1-dim.	2276.042	122	0.001				0.839	0.833	0.046	[0.041, 0.052]
6	3-dim.	530.220	431	0.001	77.918	3	0.001	0.970	0.967	0.037	[0.025, 0.047]
	1-dim.	755.989	434	0.001				0.902	0.895	0.066	[0.058, 0.073]
7	3-dim.	1078.340	899	0.001	110.370	3	0.001	0.969	0.967	0.030	[0.022, 0.036]
	1-dim.	1458.058	902	0.001				0.904	0.899	0.052	[0.047, 0.057]
8	3-dim.	696.816	524	0.001	76.199	3	0.001	0.974	0.972	0.035	[0.028, 0.042]
	1-dim.	979.228	527	0.001				0.933	0.928	0.057	[0.052, 0.063]


*df, degrees of freedom; CFI, comparative fit index; TLI, Tucker–Lewis index; RMSEA, root mean square error of approximation; χ^*2*^ and df were estimated by WLSMV. Δχ^*2*^ was estimated with the difference test procedure in MPlus (see Muthén and Muthén, 2012). C.I., confidence interval*.

**Table 4:** Goodness of fit indices for testing the dimensionality of mathematics from Grades 1 to 8.

Grade	Model	χ^2^	df	p	Δχ^2^	Δdf	p	CFI	TLI	RMSEA	90% C.I.
1	3-dim.	409.506	249	0.001	95.309	3	0.001	0.953	0.948	0.077	[0.063, 0.090]
	1-dim.	586.328	252	0.001				0.902	0.893	0.110	[0.099, 0.122]
2	3-dim.	543.407	321	0.001	96.826	3	0.001	0.944	0.939	0.061	[0.052, 0.070]
	1-dim.	734.133	324	0.001				0.897	0.889	0.083	[0.075, 0.091]
3	3-dim.	171.573	149	0.01	15.784	3	0.01	0.923	0.912	0.046	[0.000, 0.075]
	1-dim.	194.581	152	0.01				0.855	0.837	0.063	[0.032, 0.087]
4	3-dim.	236.477	206	0.01	40.265	3	0.001	0.940	0.933	0.060	[0.000, 0.093]
	1-dim.	268.352	209	0.01				0.883	0.871	0.083	[0.050, 0.111]
5	3-dim.	381.365	186	0.001	110.584	3	0.001	0.939	0.931	0.060	[0.052, 0.069]
	1-dim.	675.939	189	0.001				0.847	0.830	0.095	[0.087, 0.102]
6	3-dim.	680.214	492	0.001	112.972	3	0.001	0.912	0.906	0.054	[0.043, 0.063]
	1-dim.	966.684	495	0.001				0.780	0.765	0.085	[0.077, 0.093]
7	3-dim.	1182.063	816	0.001	205.034	3	0.001	0.968	0.966	0.047	[0.041, 0.052]
	1-dim.	1882.948	819	0.001				0.908	0.903	0.079	[0.075, 0.084]
8	3-dim.	3021.062	557	0.001	165.118	3	0.001	0.876	0.867	0.124	[0.120, 0.128]
	1-dim.	3412.642	560	0.001				0.856	0.847	0.133	[0.129, 0.137]


*df, degrees of freedom; CFI, comparative fit index; TLI, Tucker–Lewis index; RMSEA, root mean square error of approximation; χ^*2*^ and df were estimated by WLSMV. Δχ^*2*^ was estimated with the difference test procedure in MPlus (see Muthén and Muthén, 2012). C.I., confidence interval*.

**Table 5:** Goodness of fit indices for testing the dimensionality of science from Grades 1 to 8.

Grade	Model	χ^2^	df	p	Δχ^2^	Δdf	p	CFI	TLI	RMSEA	90% C.I.
1	3-dim.	596.485	461	0.001	57.623	3	0.001	0.921	0.915	0.050	[0.038, 0.061]
	1-dim.	659.870	464	0.001				0.886	0.878	0.060	[0.049, 0.073]
2	3-dim.	464.075	321	0.001	39.177	3	0.001	0.944	0.939	0.038	[0.030, 0.045]
	1-dim.	554.254	324	0.001				0.910	0.903	0.048	[0.041, 0.055]
3	3-dim.	732.349	431	0.01	66.500	3	0.01	0.924	0.918	0.111	[0.097, 0.124]
	1-dim.	786.319	434	0.01				0.911	0.904	0.119	[0.106, 0.133]
4	3-dim.	159.502	132	0.01	19.191	3	0.001	0.939	0.930	0.060	[0.000, 0.091]
	1-dim.	178.564	135	0.01				0.904	0.891	0.075	[0.041, 0.103]
5	3-dim.	571.944	402	0.001	151.940	3	0.001	0.938	0.933	0.040	[0.033, 0.048]
	1-dim.	950.437	405	0.001				0.801	0.787	0.072	[0.066, 0.078]
6	3-dim.	716.173	402	0.001	332.375	3	0.001	0.934	0.928	0.048	[0.063, 0.074]
	1-dim.	1925.098	405	0.001				0.679	0.655	0.106	[0.101, 0.111]
7	3-dim.	999.868	524	0.001	185.888	3	0.001	0.882	0.874	0.039	[0.035, 0.042]
	1-dim.	1564.230	527	0.001				0.743	0.726	0.057	[0.054, 0.060]
8	3-dim.	664.189	374	0.001	112.367	3	0.001	0.882	0.872	0.041	[0.036, 0.046]
	1-dim.	897.133	377	0.001				0.788	0.772	0.055	[0.050, 0.060]


*df, degrees of freedom; CFI, comparative fit index; TLI, Tucker–Lewis index; RMSEA, root mean square error of approximation; χ^*2*^ and df were estimated by WLSMV. Δχ^*2*^ was estimated with the difference test procedure in MPlus (see Muthén and Muthén, 2012). C.I., confidence interval*.

In most cases, the 3-dimensional models fitted the data significantly better than that the 1-dimensional models. In some cases, mostly in Grades 7 and 8, the 3-dimensional model fit indices were lower. This could have been because the tasks were originally developed for students in lower grades.

The fit indices dropped in the case of mathematics and science in Grade 8 but were significantly higher than that of the 1-dimensional model. Thus, the psychological, application and disciplinary dimensions of learning could be distinguished. The psychological dimension of learning could be made visible independently of the measured domain in everyday educational settings, thus supporting Hypothesis 1.

### The Psychological Dimension ofLearning Across Domains

The bivariate correlations of the psychological dimensions between pairs of domains (mathematics and reading, mathematics and science, and reading and science) ranged from 0.29 to 0.49 and were statistically significant (Table 6). At each grade level, the correlations of the psychological dimension (reasoning/thinking) tended to be the highest between mathematics and reading and lowest between mathematics and science. The strongest set of correlations, independent of the measured domain, was found in Grade 8, indicating that the psychological dimension of learning in reading, mathematics and science were highly correlated, but not identical constructs.

**Table 6:** Correlations of the psychological dimension between pairs of domains from Grades 1 to 8.

	Correlations of the psychological dimension		
**Grade**	**Between mathematics and reading**	**Between mathematics and science**	**Between reading and science**
1	0.426	0.372	0.407
2	0.435	0.342	0.421
3	0.390	0.289	0.340
4	0.452	0.421	0.420
5	0.436	0.404	0.429
6	0.437	0.421	0.398
7	0.440	0.429	0.395
8	0.493	0.452	0.438


*All coefficients are significant at *p* < 0.0001 level*.

The invariance in the psychological dimension of learning across the three domains was supported by comparing the 3-dimensional measurement model, which distinguishes the psychological dimension of reading, mathematics and science, and the 1-dimensional measurement model, which combines the psychological dimension of the different learning domains under a single factor. The special χ^2^-difference test in Mplus showed that the 3-dimensional model fitted significantly better at each grade level than the 1-dimensional model (Table 7).

**Table 7:** Goodness of fit indices for testing the dimensionality of the psychological dimension in reading, mathematics, and science using 1- and 3-dimensional models for Grades 1 to 8.

Grade	Model	χ^2^	df	p	Δχ^2^	Δdf	p	CFI	TLI	RMSEA	90% C.I.
1	3-dim.	406.929	321	0.001	55.558	3	0.001	0.963	0.959	0.046	[0.031, 0.059]
	1-dim.	510.320	324	0.001				0.919	0.912	0.067	[0.056, 0.078]
2	3-dim.	282.857	167	0.001	75.885	3	0.001	0.890	0.903	0.062	[0.049, 0.074]
	1-dim.	449.393	170	0.001				0.765	0.738	0.095	[0.085, 0.106]
3	3-dim.	180.800	167	0.001	20.178	3	0.001	0.921	0.910	0.036	[0.000, 0.069]
	1-dim.	203.772	170	0.001				0.806	0.783	0.056	[0.014, 0.082]
4	3-dim.	209.681	206	0.001	42.211	3	0.001	0.990	0.989	0.018	[0.000, 0.060]
	1-dim.	289.741	209	0.001				0.775	0.751	0.083	[0.058, 0.105]
5	3-dim.	398.477	296	0.001	126.509	3	0.001	0.934	0.928	0.039	[0.028, 0.049]
	1-dim.	755.052	299	0.001				0.707	0.681	0.082	[0.075, 0.089]
6	3-dim.	592.088	431	0.001	80.817	3	0.001	0.901	0.890	0.078	[0.062, 0.093]
	1-dim.	785.255	434	0.001				0.767	0.750	0.115	[0.102, 0.128]
7	3-dim.	1154.972	699	0.001	187.282	3	0.001	0.912	0.906	0.059	[0.053, 0.065]
	1-dim.	1893.066	702	0.001				0.769	0.757	0.095	[0.090, 0.100]
8	3-dim.	471.630	347	0.001	142.432	3	0.001	0.918	0.911	0.042	[0.031, 0.059]
	1-dim.	747.482	350	0.001				0.740	0.719	0.072	[0.065, 0.079]


*df, degrees of freedom; CFI, comparative fit index; TLI, Tucker–Lewis index; RMSEA, root mean square error of approximation; χ^*2*^ and df were estimated by WLSMV. Δχ^*2*^ was estimated with the difference test procedure in MPlus (see Muthén and Muthén, 2012). C.I., confidence interval*.

### The Rate of Development in the Psychological Dimension

**FIGURE 3:**
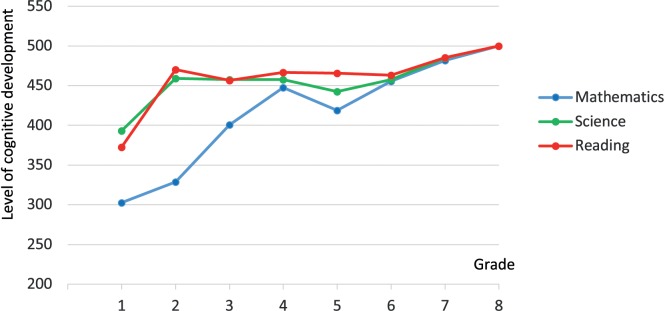
The speed of the cognitive development in the psychological dimension of learning within the domains of mathematics, science and reading (Please note, that in each measured domain the mean of the 8th graders’ achievement was artificially set to 500 with a standard deviation of 100).

Figure 3 presents the mean cognitive development scale scores in the psychological dimension of learning reading, mathematics and science. Please note that in each domain, the mean score of Grade 8 students was set at 500 with a standard deviation of 100, thereby constructing the point of reference for interpreting students’ achievement. This means that we cannot compare the development of the psychological dimension of learning across domains, but we can compare the rate of development.

We found that the amount and rate of cognitive development were almost the same in each domain between Grades 6 and 8 and that there was no appreciable development in reading and science between Grades 2 and 6. The greatest rate of progress occurred in Grade 1 in reading and science, but not mathematics. Generally, there was a steady increase in the psychological dimension of learning in mathematics, especially in the first 4 years of schooling. The results confirmed our hypothesis that children’s cognitive development is slow (Molnár et al., 2013; Molnár et al., 2017), thus indicating the importance of the explicit development in this dimension in school lessons. Overall, these results highlighted the importance, sensitivity and potential of the development of thinking skills in the early years of schooling.

### Relationship Between the Three Dimension of Learning

The possibility and practical relevance of separating the psychological dimension of learning can be explored from another perspective by examining the proportion of its variance that remains unexplained if the more readily visible disciplinary and application dimensions (referred to together as school knowledge) are taken into account. Technically, these dimensions may be considered as potential predictors of the psychological dimension.

**FIGURE 4:**
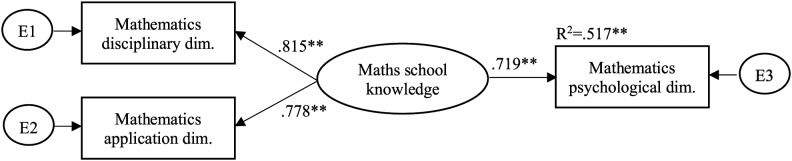
A structural model of mathematics school knowledge as a predictor of students’ cognitive development in the domain of mathematical reasoning.

**FIGURE 5:**
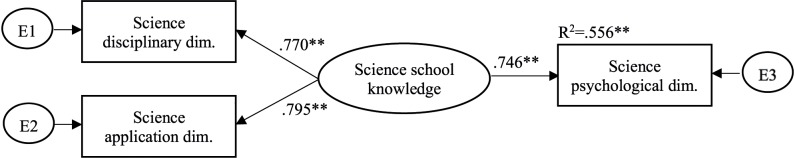
A structural model of science school knowledge as a predictor of students’ cognitive development in the domain of scientific reasoning.

**FIGURE 6:**
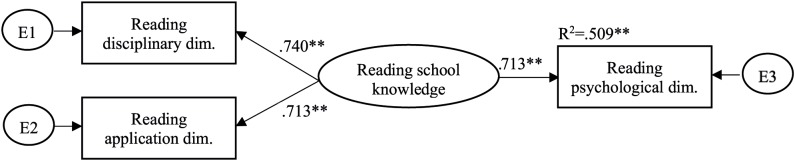
A structural model of reading school knowledge as a predictor of students’ cognitive development in the domain of the psychological dimension of reading.

We used continuous factor indicators in SEM analyses to examine the relationships between school knowledge and the psychological dimension of learning in each domain. School knowledge as a latent factor was specified as the application and disciplinary dimensions of learning. According to the results, school knowledge predicted the psychological dimension of learning in all domains, but a significant amount of variance remained unexplained (see Figure 4–6). This indicates that existing aspects of the psychological dimension of learning can be separated from school knowledge as measured by the disciplinary and application parts of students’ knowledge. That is, it is relevant to measure the psychological dimension of learning in addition to measuring the disciplinary and application dimensions of learning. So our hypothesis was confirmed.

The amount of explained variance was statistically significant and almost the same for mathematics and reading and somewhat higher for science. This suggests that there may be more common reasoning aspects in the three dimensions of science. The model for each domain fitted well (CFI = 1.000, TLI = 1.000, RMSEA = 0.000).

### Gender Difference in the Psychological Dimension of Learning

In the present study, girls outperformed boys in the psychological dimension of learning in reading, mathematics and science (Mathematics: *F* = 0.272, *t* = -6.696, *p* < 0.001; Science: *F* = 3.578, *t* = -11.525, *p* < 0.001; Reading: *F* = 3.224, *t* = -4,370, *p* < 0.001); however, this varied by grade level (see Table 8). The largest, statistically significant differences in favor of girls were found in Grades 4 and 5, where girls outperformed boys in all three domains, and in Grades 6 to 8, where girls outperformed boys in two of the three domains. Girls also outperformed boys in reading in Grades 3–8, in mathematics in Grades 1 and 4–6, and in science in Grades 1, 4, 5, 7, and 8.

**Table 8:** Gender differences in the psychological dimension of learning in reading, mathematics and science in Grades 1 to 8.

Grade	Area	N	Boys’ mean (SD)	Girls’ mean (SD)	F	p	t	p	d
1	R	685	346 (128)	359 (123)	0.075	0.784	-1.404	0.161	0.107
	M	707	306 (117)	332 (129)	2.058	0.152	-2.871	0.004	0.215
	S	487	371 (98)	392 (121)	7.794	0.005	-2.143	0.033	0.030
2	R	1024	457 (113)	462 (118)	0.387	0.534	-0.705	0.481	0.044
	M	1033	338 (137)	338 (144)	0.252	0.616	-0.031	0.975	0.000
	S	668	444 (100)	456 (102)	0.258	0.612	-1.505	0.133	0.118
3	R	1152	439 (126)	463 (123)	0.736	0.391	-3.214	0.001	0.192
	M	1236	420 (103)	417 (109)	2.389	0.122	0.465	0.642	0.028
	S	829	455 (89)	460 (93)	0.227	0.634	-0.728	0.467	0.054
4	R	1539	452 (107)	473 (106)	0.237	0.627	-3.839	0.000	0.197
	M	1567	451 (99)	465 (101)	0.638	0.425	-2.832	0.005	0.139
	S	862	443 (109)	463 (100)	2.178	0.140	-2.772	0.006	0.191
5	R	1721	447 (113)	479 (104)	3.374	0.066	-6.209	0.000	0.294
	M	1877	422 (95)	439 (100)	1.849	0.174	-3.798	0.000	0.174
	S	1540	429 (104)	453 (96)	4.063	0.044	-4.725	0.000	0.239
6	R	1559	445 (105)	480 (99)	1.466	0.226	-6.858	0.000	0.342
	M	1496	460 (89)	469 (96)	1.991	0.158	-2.035	0.042	0.097
	S	1469	452 (111)	457 (109)	0.113	0.737	-0.842	0.400	0.045
7	R	1280	465 (107)	490 (97)	2.239	0.135	-4.341	0.000	0.244
	M	1291	481 (99)	491 (88)	4.528	0.034	-1.859	0.063	0.106
	S	1250	480 (98)	493 (96)	0.100	0.752	-2.259	0.024	0.134
8	R	1035	481 (101)	515 (96)	3.165	0.076	-5.429	0.000	0.345
	M	932	494 (102)	505 (96)	1.703	0.192	-1.749	0.081	0.111
	S	954	490 (99)	509 (98)	0.014	0.906	-2.958	0.003	0.192


*R, reading; M, mathematics; S, science; F, *F*-value; t, *t*-value; p, significance level; d, Cohen-d.*

In this section, we examine gender differences among Grade 8 students – the grade level of students in PISA, TIMSS and our study. The results confirm our hypotheses that an assessment which focuses on students’ disciplinary knowledge or application does not replace an assessment of the psychological dimension of learning. In the case of mathematics, no gender differences were detected in the application and psychological dimensions of learning, but girls scored significantly higher, on average, than boys in the disciplinary dimension of learning. The results were different in the case of science. There were no gender differences in the application dimension of science learning. Boys achieved significantly higher in the psychological dimension.

## Discussion

Previous research has already identified several characteristics of learning reading, mathematics and science. However, it has mainly focussed on only one dimension; either the disciplinary dimension or the application dimension of learning, and on the reading, mathematics and science learning of older students. There have been significant attempts to concentrate on the application and reasoning dimensions, but educational practice has mostly focussed on the assessment of the content of individual curriculum subjects. The application of knowledge has seldom been assessed, although the PISA assessments have highlighted its importance. Because of the lack of easy-to-use assessments, the psychological dimension of learning (cognitive development and reasoning) remains hidden. Therefore, neither the students nor their teachers receive feedback on level or development in this dimension. This study provides evidence that the psychological dimension of learning can be made visible and that technology-based assessment may be applied in an everyday educational context. This evidence highlights the importance of the assessment and the explicit development of the psychological dimension of learning in a school context. Further, it points to gender differences in the developmental rate of the psychological dimension of learning in favor of girls, although this varies by grade and domain.

Results support our hypotheses that the three dimensions of learning can be distinguished empirically and can be assessed separately. The 3-dimensional frameworks derived from previous research, including international comparative studies (Csapó and Szendrei, 2011; Csapó and Csépe, 2012; Csapó and Szabó, 2012), showed relatively good validity, and the results from the current analyses confirmed that they may form evidence-based foundations for diagnostic assessment. The most important findings from these analyses was that the psychological dimension of learning can be measured at the primary school level in the context of three of the most important domains of learning – reading, mathematics and science.

The present results also confirmed that, although the roots of the psychological development of different domains are universal and the domains of learning build on each other (Molnár and Csapó, 2019), there are still significant developmental differences between them. While there is a close connection between the development of early literacy and numeracy, and later mathematics learning builds on reading, and science builds on both (McKeachie, 1987), our results support the notion that the transfer is not obvious between the different domain contexts. There were statistically significant correlations between the development scores in the psychological dimension of reading, mathematics and science learning, but they were not identical constructs.

Previous studies have indicated that children’s cognitive development is slow (Molnár et al., 2013, 2017) but that it can be taught effectively (de Koning et al., 2002; Klauer and Phye, 2008; Perret, 2015). Our results confirmed both of these notions as there was no appreciable development in the psychological dimension of learning in reading and science for students in Grades 2–6, and students’ cognitive development was the most steady (and effective) in mathematics, where the greatest development took place in the first years of schooling. This confirms previous research findings and highlights the potential of developing thinking skills in the early years of schooling.

The results of the SEM indicated the complex nature of learning in reading, mathematics and science. An examination of the predictive power of school knowledge on the psychological dimension of learning showed that the disciplinary and application dimensions of learning together predicted the psychological dimension of learning at a moderate, but statistically significant level, while a significant amount of variance remained unexplained. This indicates that school knowledge in reading, mathematics, and science can contribute to the development of the psychological dimension of learning and can stimulate students’ general cognitive development, but the transfer effect may not be high. The results suggest that aspects of the psychological dimension of learning exist and can be separated from the learning dimensions assessed most often at school and in international comparative studies. This highlights the importance and relevance of developing measures of the psychological dimension of learning as well.

To provide context to interpret the size of the gender difference in the psychological dimension of learning in reading, mathematics and science, we compared our results to findings on gender differences in the most prominent international comparative studies. The gender differences in the international studies at Grade 4 and 8 were found in our study. We found gender differences in reading over almost all the primary school grade levels, including Grades 4 and 8, indicating that girls perform better in reading, irrespective of the dimension of learning.

## Limitations of the Present Study

As the PISA 2015, TIMSS 2015, and PIRLS 2016 studies have also indicated, there are large differences between countries not only on the level of reading, mathematics and science performance, but also in gender differences. Therefore, results found in one country cannot be generalized across countries and cultures. Although general trends have been found, the generalizability of the results may be limited. The method we applied in this study was generalizable and may be useful for making the psychological dimension of learning visible in any educational context. A further limitation of the study could be the results of the “common method bias” and “test motivation” as possible sources of shared variance across tests and domains. Participation in the study was voluntary, and although the large sample sizes and the diversity of the schools made the results sufficiently robust, the actual samples were not nationally representative. Thus, the present study does not provide a complete picture of the Hungarian education system. Nevertheless, the analyses did reveal some generalizable trends.

## Conclusion

The 3-dimensional frameworks for the diagnostic assessment used in the present study were devised on the basis of current results from a number of research fields ranging from cognitive neuroscience to research on cognitive development, standard setting and the theoretical frameworks of large-scale international comparative studies. The item banks for assessing reading, mathematics and science were developed through the careful mapping of assessment tasks onto frameworks. The next step in scientifically establishing and further developing the diagnostic system is to empirically validate the 3-dimensional framework. We first presented the results of the comprehensive analyses in this study. In the present analyses, we focused on the psychological dimension of learning, which determines the dimensions of disciplinary knowledge and application, but is less visible or observable in the school context.

The results confirmed the theoretical foundations of the project and made clear that the psychological dimension can be distinguished and measured in the context of the most important domains of learning in the beginning phase of schooling. These findings indicate directions for further research as well. Item development for this study was based on the theoretical frameworks without empirical evidence of dimensionality. Based on the empirical confirmation of the three dimensions in this study, the validity of the assessment scales constructed from the item banks, may be improved by exploring how well the items fit particular scales.

Establishing scales empirically to assess the psychological dimension of learning paves the way to improving learning as well. The evidence that cognitive development is measurable provides a basis for large-scale systematic diagnostic monitoring of the development of students’ thinking skills, one of the most sorely lacking elements in the current spectrum of assessment practices. It also supports different types of intervention studies from teacher-initiated practical improvements to well-controlled, randomized experiments.

## Ethics Statement

The authors only had access to anonymized data, and hence an ethics approval and parental consent were not required as per applicable institutional and national guidelines and regulations. The assessment data collected for this study formed integrated parts of the normal educational processes of the participating schools. The coding system for the online platform masked students’ identity, the researchers would thus have been unable to tie the data to the students. The results from the low-stakes diagnostic assessments were only disclosed to the participating students (as immediate feedback) and to their teachers. Because of the anonymity and low-stakes testing design of the assessment process, it was not required or possible to request and obtain written informed parental consent from the participants.

## Author Contributions

GM and BC took responsibility for the content, including participation in the concept, design, analysis, drafting the manuscript, writing and final approval of the manuscript, and agreed to be accountable for all aspects of the study.

## Conflict of Interest Statement

The authors declare that the research was conducted in the absence of any commercial or financial relationships that could be construed as a potential conflict of interest.
